# Patient roadmap and economic burden of chronic tick-borne illness and post-treatment Lyme disease syndrome in Ireland, and public health issues arising

**DOI:** 10.3389/fpubh.2026.1667043

**Published:** 2026-06-10

**Authors:** Gordana Avramovic, Leona Gilbert, Sławomir Kujawski, Sigrid Blehle, Pascal Jollivet-Courtois, Paweł Zalewski, Nabin Adhikari, Kunal Garg, John Shearer Lambert

**Affiliations:** 1 Department of Infectious Diseases, Catherine Mc Auley Education & Research Centre, Mater Misericordiae University Hospital, Dublin, Ireland; 2 Catherine Mc Auley Education & Research Centre, University College Dublin, Dublin, Ireland; 3 Tezted Oy, Jyväskylä, Finland; 4 Department of Exercise Physiology and Functional Anatomy, Ludwik Rydygier Collegium Medicum in Bydgoszcz, Nicolaus Copernicus University in Toruń, Bydgoszcz, Poland; 5 Privatpraxis für Lyme/Borreliose und Chronische Infektionen—Alviasana, Augsburg, Germany; 6 Université de Technologie de Compiègne, Compiègne, France; 7 Laboratory of Centre for Preclinical Research, Department of Experimental and Clinical Physiology, Warsaw Medical University, Warsaw, Poland

**Keywords:** chronic Lyme disease, cost, Lyme, patient roadmap, post-treatment Lyme disease syndrome, tick-borne infections

## Abstract

Lyme borreliosis (LB), commonly referred to as Lyme disease (LD), is a prominent global health issue, exhibiting a seroprevalence rate of 14.5%. Heightened incidence levels of LD have been recorded in parts of Europe, Poland, Eastern Europe, and the Baltic States. The research aimed to inform the cost of LD and post-treatment Lyme disease syndrome (PTLDS) in Ireland through results from a patient questionnaire, disease modelling, the construction of a patient roadmap, and attempts to arrive at prevalence calculation estimates based on local data. Patient data encompassed sociodemographic particulars, disease attributes, healthcare resource utilization, and the influence on their employment status. Of 301 patients, 210 were diagnosed with LD and/or a tick-borne infection (TBI), the cohort’s average age was 40.07 (SD 13.5) (*N* = 210; Female:Male 60:40). The mean duration of symptoms in PTLDS patients was 7.15 years. The average number of visits to other healthcare professionals was 16.8 per patient. Regarding current employment status, the data indicates that 50.2% of respondents were currently working, 10.1% were unemployed, 8.7% were retired, 5.3% had caring responsibilities, 11.1% were on sick leave, and 14.5% fell into the “Other” category. Additionally, when asked if symptoms had affected their employment status, 69% of respondents said yes, 26% said no, and 5% did not respond. Modeling efforts show that the roadmap to care for PTLDS is challenging, leading to wandering from specialty to specialty and high healthcare utilization. Utilizing a novel method of indirect reverse estimation, our lifetime risk or cumulative incidence of PTLDS estimation is at 0.003%. Lack of data collection from Irish health authorities is leaving the issue of the cost of LD and PTLDS hard to address, despite efforts from our single-site study.

## 1 Introduction

The bacterium *Borrelia burgdorferi sensu lato* group (*Borrelia*) is the cause of Lyme borreliosis (LB), also commonly known as Lyme disease (LD). LD has emerged as a significant health concern in Europe and worldwide, with global seroprevalence at 14.5% (1). In Europe, higher seroprevalences (2) have been recorded up to 54.9% in Poland, Eastern Europe (3–5), and 46.7% in the Baltic States (5, 6). Unfortunately, national surveillance systems often underreport the true number of LD cases. These systems rely on passive reporting, which means many cases are missed, whether because patients are underdiagnosed, symptoms are misclassified, physicians report inconsistently, or only the classic erythema migrans (EM) rash is recognized. As a result, the incidence figures generated from surveillance data may not provide an accurate picture of the real burden of disease in the community (4). Incidence figures for LD across Europe need to be interpreted with care because surveillance systems are not consistent from country to country. Some countries count both EM and disseminated LD cases, while others report only Lyme neuroborreliosis (LNB). As Burn et al. (5) have shown, these differences in case definitions and reporting practices explain much of the variation seen across Europe. For instance, countries that apply broader definitions, such as Estonia, Lithuania, Slovenia, and Switzerland, report more than 100 cases per 100,000 person-years, while those with narrower definitions, including Ireland and several Western European countries, report fewer than 20 per 100,000 person-years (2, 4, 5). Even within countries, rates can vary: in certain regions of Belgium, the Czech Republic, France, Germany, and Poland, incidence exceeds 100 per 100,000. These differences show that surveillance methods, as much as the underlying epidemiology, shape the numbers we see.

Moreover, a significant proportion of LD patients, 20–27% (7–11), who receive the recommended therapy (12) experience long-term repercussions of the condition known as “post-treatment Lyme disease syndrome” (PTLDS) or “chronic Lyme disease” (CLD). PTLDS refers to persistent or recurring symptoms lasting more than 6 months after completing the recommended antibiotic treatment (7, 9, 13–15). For the purpose of this article, we use the term PTLDS consistently, as all participants in our cohort met strict clinical criteria for this definition (persistent symptoms >6 months post-treatment). While the broader literature sometimes uses ‘Chronic Lyme Disease’ (CLD) interchangeably, CLD encompasses a more heterogeneous group that includes untreated or partially treated patients. Our study specifically isolates the PTLDS phenotype to align with guideline-based definitions and enable precise cost and care-pathway modeling. Moreover, the US Centers for Disease Control and Prevention (CDC) has officially recognized the medical significance of persistent symptoms after acute Lyme disease, and has included these in the federal framework for Infection-Associated Chronic Conditions and Illnesses (IACCIs), recognizing the chronic condition arising from Lyme Disease Infection (16). Chronic symptoms may include fatigue, musculoskeletal discomfort, and cognitive impairment. Precise case numbers remain unknown, but modeling studies estimate the prevalence of PTLDS in the United States to be as high as 2 million individuals (9, 17). Regrettably, these statistics are likely underestimated, in part because patients without the early-localized EM rash are often missed at the initial stage and may only be diagnosed once disseminated manifestations such as Lyme neuroborreliosis, Lyme carditis, or Lyme arthritis appear. If diagnosis or treatment is delayed or unsuccessful at these stages, some patients may then go on to develop persistent symptoms consistent with PTLDS (14, 18). Many available studies tend to confirm that, in a larger number of cases, the initial infection may go clinically unnoticed and, therefore, receive no treatment (19–22). There is scientific disagreement in the literature and between guidelines (e.g., ILADS vs. IDSA) regarding whether persistent symptoms represent ongoing infection or post-infectious sequelae, but in line with CDC and WHO ICD standards, this paper will use the term PTLDS. Regardless of etiology, patients experience disabling symptoms and incur substantial healthcare and societal costs.

Therefore, there are significant challenges in the assessment of the cost of PTLDS to the Irish healthcare system. Such cost implications for PTLDS have not been studied extensively by Irish authorities. There is no comprehensive data based on solid epidemiological evidence across the range of the manifestation of LD and co-infections, inclusive of PTLDS, and our study is offering to bridge some of the gaps in this area, where possible, and as seen from the perspective of a single clinic. Our article offers some real-world data based on patient results from a tick-borne infection (TBI) clinic and, as such, brings some more concrete data to the issue in Ireland. This study is intentionally exploratory and designed as a preliminary proof-of-concept to integrate patient-reported care pathways, direct consultation costs, and indirect prevalence modeling in a setting lacking national surveillance data. Each component (patient roadmap, economic burden, and prevalence estimation) warrants dedicated, large-scale investigation; however, given the absence of Irish-specific data, this single-clinic cohort provides a foundational baseline for future multi-center epidemiological and health-economic research.

Although Irish-specific data on the cost of Lyme disease (LD) and PTLDS are lacking, figures from other countries highlight the scale of the economic burden. Governments allocate up to 20 billion EUR to manage affected individuals (23, 24). In Belgium and the Netherlands, the estimated annual financial burden for patients with LD is 5.59 million EUR, but for those with PTLDS, the cost rises dramatically to about 149.8 million EUR—26.7 times higher (25, 26). Beyond these national examples, six studies (27) have evaluated the broader economic impact of LD from a societal perspective. Reported yearly costs include 735,550 USD in Scotland, 142,562 USD in Sweden, 40.88 million USD in Germany, 23.12 million USD in the Netherlands, and up to 786 million USD in the USA. These calculations, as emphasized in several studies (26, 28, 29), demonstrate the substantial financial burden associated with LD alone, without yet accounting for PTLDS. For instance, Hook and colleagues (30) estimated the total yearly cost of diagnosed LD in the United States at 345–968 million USD. Taken together, these findings indicate that while estimates differ by country and methodology, both LD and PTLDS represent considerable healthcare and societal costs. Importantly, the growing recognition of this complex disease is reflected in the increasing attention it has received in recent years (11, 31–33), along with greater acknowledgement of its public health impact (16, 34). These calculations, as demonstrated in many studies (26, 28, 29), have emphasized the substantial financial burden associated with LD alone without considering the potential effects of PTLDS. For instance, the total yearly cost of diagnosed LD in the US is estimated to be around 345–968 M USD, according to Hook and colleagues (30). Still, the complex disease is gaining proper attention (11, 31–33) with growing recognition and status over time (16, 34).

While most epidemiological and clinical research focuses on Lyme disease (LD) or post-treatment Lyme disease syndrome (PTLDS) in isolation, it is well established that *Ixodes* ticks frequently transmit multiple pathogens concurrently. Co-infections (e.g., *Babesia*, *Anaplasma*, *Bartonella*, and *Rickettsia* spp.) can significantly increase symptom severity, prolong diagnostic delays, and complicate clinical management (7, 35–40). In routine practice, patients frequently present with polymicrobial tick-borne infections rather than isolated *Borrelia burgdorferi* exposure. Accordingly, the chronic patient cohort in this study includes individuals diagnosed with PTLDS, those with chronic tick-borne co-infections independent of confirmed LD, and patients with overlapping PTLDS and co-infection presentations. This inclusive approach reflects the real-world clinical complexity of tick-borne diseases in Ireland, where symptom heterogeneity and fragmented care pathways often obscure the distinction between isolated and polymicrobial chronic manifestations.

Making the diagnosis of the disease is clinically difficult, as described in a report from Quebec’s National Institute for Excellence in Health and Social Services (INESSS) (41). Caught between the scientific debates, for instance, the discrepancies between ILADS (International Lyme and Associated Disease Society) and IDSA (Infectious Disease Society of America) guidelines, the lack of solid reporting surrounding LD and TBIs, and the lack of scientific resolution about how to deal with chronic symptoms, patients are left without a clear roadmap to care. In many medical specialties, patient roadmaps are crucial because they offer structured frameworks to support decision-making, consider patient preferences, enhance safety, and optimize healthcare outcomes. To ensure that patient values are the basis for healthcare decisions, research methodologies are designed to align with patient needs, and this is achieved through frameworks such as the ISPOR (the Professional Society for Health Economics and Outcomes Research) roadmap for incorporating patient preferences into decision-making (42). Roadmaps have been used in many areas of healthcare to guide research, treatment, and policy. For example, Kowalski et al. (43) proposed a strategy for preclinical autoimmunity in rheumatoid arthritis, while Stegemann et al. (44) emphasized patient-centred methods in drug development to improve usability and adherence. Wilson et al. (45) demonstrated how an implementation roadmap for Unique Device Identification can enhance patient safety, and Ward et al. (46) showed how visual roadmaps can assess cost-effectiveness in populations at risk for Hepatitis C. Building on these approaches, our article introduces the first patient roadmap for PTLDS, capturing patients’ journeys through the healthcare system and supporting future research into costs and care utilization. This study represents the first attempt in Ireland to model the PTLDS burden using patient journey mapping and indirect prevalence estimation, bridging critical epidemiological and health-economic data gaps.

## 2 Methods

### 2.1 Study population and recruitment

All patients (both sexes) over 16 years of age who received consultations at the outpatient infectious diseases clinic at The Mater Misericordiae University Hospital, Ireland, were invited to participate in this study due to suspected tick-borne infections between December 2019 and January 2022. Of the 301 patients evaluated by the consultant at the initial visit, 210 were clinically diagnosed with tick-borne infection (TBI). These patients had non-specific symptoms resembling the flu and a positive history of tick bites or bull’s eye rash, raising the suspicion of TBIs, and/or had positive serology results for one or more TBIs (11). All 210 patients included in the final analytical cohort strictly met the clinical definition of PTLDS. PTLDS was defined as persistent or recurring symptoms lasting >6 months following completion of guideline-recommended antibiotic therapy for confirmed Lyme disease. Individuals with untreated chronic Lyme disease (CLD), isolated tick-borne co-infections without prior *Borrelia*-directed treatment, or alternative non-tick-borne etiologies were excluded. This strict inclusion criterion ensures that our sample accurately represents the PTLDS phenotype as defined by prevailing clinical guidelines, and terminology throughout the manuscript has been standardized to reflect this uniform cohort definition.

### 2.2 Ethical approvals

This study complies with the EU CT Directive 2001/20/EC, GCP Commission Directive 2005/28/EC, ICH/GCP, Declaration of Helsinki (1996 Version), and all other applicable local and international regulatory requirements. Ethics approval for the study protocol was granted by the Institutional Review Board of the Mater Misericordiae University Hospital with Institutional Review Board Reference number 1/378/1946. Informed consent was obtained from the human participants of this study under study reference 1/378/1946 from the Mater Misericordiae University Hospital.

### 2.3 Data collection

Patients were asked to fill out a questionnaire after they consented to participate in the study (11, 33). Questions related to (i) sociodemographic information (i.e., sex, age, place of residence, and country/county where the initial infection was contracted if known), (ii) disease characteristics (some risk factors including recall of a tick bite, date of the tick-bite if known, prior diagnoses associated with symptoms, and length of disease), (iii) healthcare resource utilization (prior diagnoses, prior medical visits associated with the symptoms) and (iv) impact on work situation (i.e., employment status and capacity to work).

### 2.4 Disease modelling and patient roadmap

A disease progression model and a model of the patient roadmap were constructed based on existing models used in cost-effectiveness analyses (46). Stages of the patient roadmap were constructed for PTLDS and co-infections based on existing literature and expert input from the treating team in Ireland and reviewed by an international panel of experts from Poland, Germany, and Finland for accuracy. Each stage of the roadmap can be traced back to publications from the scientific literature, and references will be highlighted in the results section. Moreover, some of the stages were illustrated further through the data obtained by our study.

### 2.5 Cost categories: data sources and calculation of costs

Direct medical costs to patients were calculated by applying unit costs to different types of healthcare resource utilization. Unit costs were obtained from the Mater Misericordiae University Hospital related to GPs and medical specialists. The average cost of a GP consultation was estimated at 60 EUR, while an average specialist/consultant consultation was estimated at 300 EUR. An out-of-pocket average cost of 180 EUR for a 45-min consultation with a GP or a specialist was derived. This average cost was used for all consultations, as no difference between GP and consultant consultations was made in the questionnaire to patients. The cost of patients’ wandering for their care was estimated based on the average number of doctors seen, as declared by patients, multiplied by the average consultation price. Any additional costs for testing when it was done privately were not calculated. Alternative therapy costs were also not calculated. In Ireland, reimbursement for consultations depends on the patient’s capacity to acquire and pay for private insurance. Private insurance reimbursements were not calculated because reimbursements vary depending on the type of insurance subscribed to and because some patients do not have any private insurance. Cost calculations reflect direct out-of-pocket consultation fees only. Indirect costs (productivity loss, travel, informal care), comorbidity-related expenditures, and probabilistic sensitivity analyses were excluded due to data unavailability. This constitutes a preliminary direct-cost approximation. Antibiotic cost estimates in Table 1 reflect drug acquisition costs only and are not weighted by individual treatment duration; therefore, average cost per patient was not calculated.

**Table 1:** Cost of antibiotic treatment and prescription frequency among PTLDS cohort (*N* = 210).

Antibiotic	Dosage	1 month (€)	2 months (€)	3 months (€)	% of cohort prescribed
Doxycycline	100 mg bd	9.77	17.64	25.07	42
Clarithromycin	500 mg bd	6.12	11.40	16.69	17
Rifampicin	300 mg od	5.85	10.21	14.99	26
Azithromycin	500 mg od 5 days a week	8.63	16.18	24.33	13
Amoxicillin	1 g tds	1.28	1.71	3.24	3
Average		31.65	57.15	84.32	

Bd = twice a day, od = once a day, tds = three times a day. Supplementary Table 1 provides data on antibiotics taken by patients. Values represent the percentage of patients prescribed each antibiotic. Antibiotic costs are provided for informational purposes only; the primary economic analysis focuses on direct out-of-pocket consultation fees (see Methods 2.5). Average antibiotic cost per patient was not calculated due to variable treatment durations and lack of individual prescription-level duration data.

### 2.6 Prevalence estimation and lifetime risk of cumulative incidence assessment

Two methodologies were employed to attempt to determine the burden of PTLDS in Ireland and to update figures found in the literature based on actual data, wherever possible, rather than projections issued from other sources. The first method applied the following standard epidemiological relationship: Prevalence = incidence rate x average duration of disease (47). It involved calculating the incidence rate by considering the number of cases per 100,000 population per year (PPY) reported in prior research (5, 40, 48) and the average length of the condition. The second method proposes a reverse, indirect estimating approach to calculate the risk of cumulative incidence. It models the pathway from tick exposure to PTLDS. The second approach used published data on tick bite exposure rates, *Borrelia* infection prevalence in ticks, the probability of developing Lyme disease after an infected tick bite, and the proportion of Lyme disease patients who develop PTLDS. Where data for Ireland were unavailable, estimates from the literature were used to fill gaps, ensuring conservative assumptions throughout. There are published data (49) on the overall rate of *Borrelial* infection in the tick population in Ireland and the percentage of patients who develop chronic Lyme disease after experiencing an initial acute episode. Indirect estimation was used to determine the prevalence of PTLDS based on these calculations.

Brestrich and colleagues calculated that the incidence rate of acute LD in Ireland is 9.5 per 100,000 PPY (49). Assuming the initial value to be 9.5, it would decrease to 0.57 per 100,000 individuals when considering the occurrence of PTLDS, considering that 6% develop PTLDS from LD. Subsequently, a significant proportion of individuals, ranging from 2027%, experience PTLDS, according to multiple studies (7–10, 50). In one comparative study, chronic symptom prevalence was 27.2% in LD patients versus 21.2% in matched controls (10). The difference (6%) represents the excess symptom burden potentially attributable to prior Borrelia exposure. Given the heterogeneity of symptom definitions and the conservative modeling approach of this study, we applied the 6% excess-risk estimate to calculate lower-bound prevalence projections. This value is explicitly noted as a conservative parameter; actual PTLDS development rates in clinical practice are likely higher (10–20%) as supported by broader literature. The prevalence of *Borrelial* infection in ticks in Ireland was assumed to be 5%, based on existing estimates (50). The risk of developing Lyme disease (LD) symptoms after a bite from an infected tick ranges from 6.7 to 14.4%, influenced by factors such as tick engorgement and attachment duration (51). For estimation, a 10% risk for developing LD after being bitten by an infected tick was used. The prevalence of post-treatment Lyme disease syndrome (PTLDS) was set at 6% among LD patients (10). Using these assumptions, calculations were performed to estimate the prevalence of LD, PTLDS, and tick bite exposure in the population. Indirect reverse estimation was applied to approximate the number of individuals bitten by ticks based on PTLDS prevalence data (52).

### 2.7 Data analysis

Data was entered in an Excel spreadsheet and transferred to SPSS version 27 for further analysis. The Sankey diagram, which has edges directly proportionate to the number of patients, was generated using RAWGraphs 2.0 beta, a software developed by Mauri et al. (53).

## 3 Results

### 3.1 Description of cohort

Of 301 patients, 210 were diagnosed with a TBI. For the LD and TBI patients, the cohort’s average age was 40.07 (SD 13.5 95% CI: 38.2–41.9), (*N* = 210; Female:Male 60:40). The final analytical cohort (*N* = 210) consisted exclusively of patients who met strict PTLDS criteria (persistent or recurring symptoms >6 months post-treatment). The mean duration of symptoms in this PTLDS cohort was 7.15 years (95% CI: 6.0–8.3). Data on the number of physicians they had seen for their condition before attending the clinic was provided by 194 patients. The average number of visits to other healthcare professionals was 16.8 per patient (95% CI: 13.6–19.9). Regarding current employment status, the data indicates that 50.2% of respondents were currently working, 10.1% were unemployed, 8.7% were retired, 5.3% had caring responsibilities, 11.1% were on sick leave, and 14.5% fell into the “Other” category. Additionally, when asked if symptoms have affected their employment status, 69% of respondents said yes, 26% said no, and 5% did not respond. Although the study cohort included patients with clinically confirmed tick-borne co-infections, detailed stratification and subgroup analysis of co-infection prevalence, symptom trajectories, and associated costs were beyond the scope of this manuscript. Co-infection data were collected but not independently analyzed for this report; dedicated analyses of polymicrobial tick-borne infections will be addressed in future publications. The care and treatment of patients for their symptoms prior to accessing the clinic was rated at an average mark of 3 out of 10, where 1 is poor, and 10 is very good. Out of 170 patients who declared they had gone to their GP for their symptoms, only 24 (14.11%) declared being satisfied with the outcome of their visit. Of 160 who declared they had gone to a specialist/consultant for their symptoms, only 22 (13.75%) declared being satisfied after their visit. The average out-of-pocket cost for physician consultations prior to specialist referral was estimated at 3,060 EUR per patient (17 × 180 EUR). Consistent with Methods 2.5, costs for private testing, travel, and productivity loss were not captured or calculated due to data unavailability. Detailed antibiotic utilization and cost data are provided in Supplementary Table 1. Costs for private testing, travel, and productivity loss were not captured per Methods 2.5.

### 3.2 Disease modeling and patient roadmap

**Figure 1:**
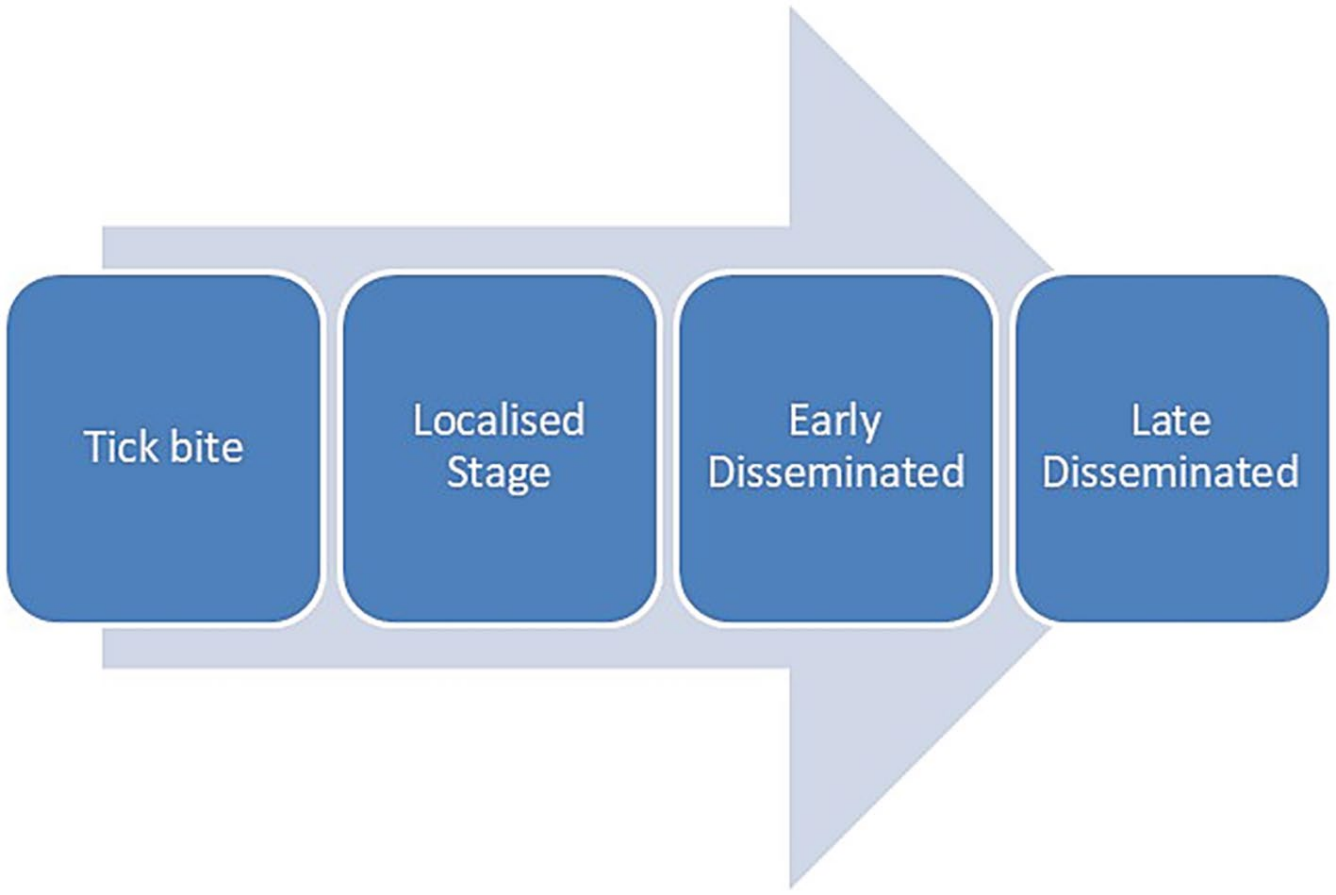
Natural history and clinical stages of Lyme borreliosis preceding chronic sequelae. The model illustrates localized, early disseminated, and late disseminated stages of untreated or delayed-diagnosis *Borrelia* infection, which may progress to persistent symptom complexes (PTLDS/CTBI) following treatment or prolonged infection. This model synthesizes established literature on Lyme borreliosis natural history (18, 39) and is presented to provide clinical context for the patient care pathways analyzed in this study.

**Figure 2:**
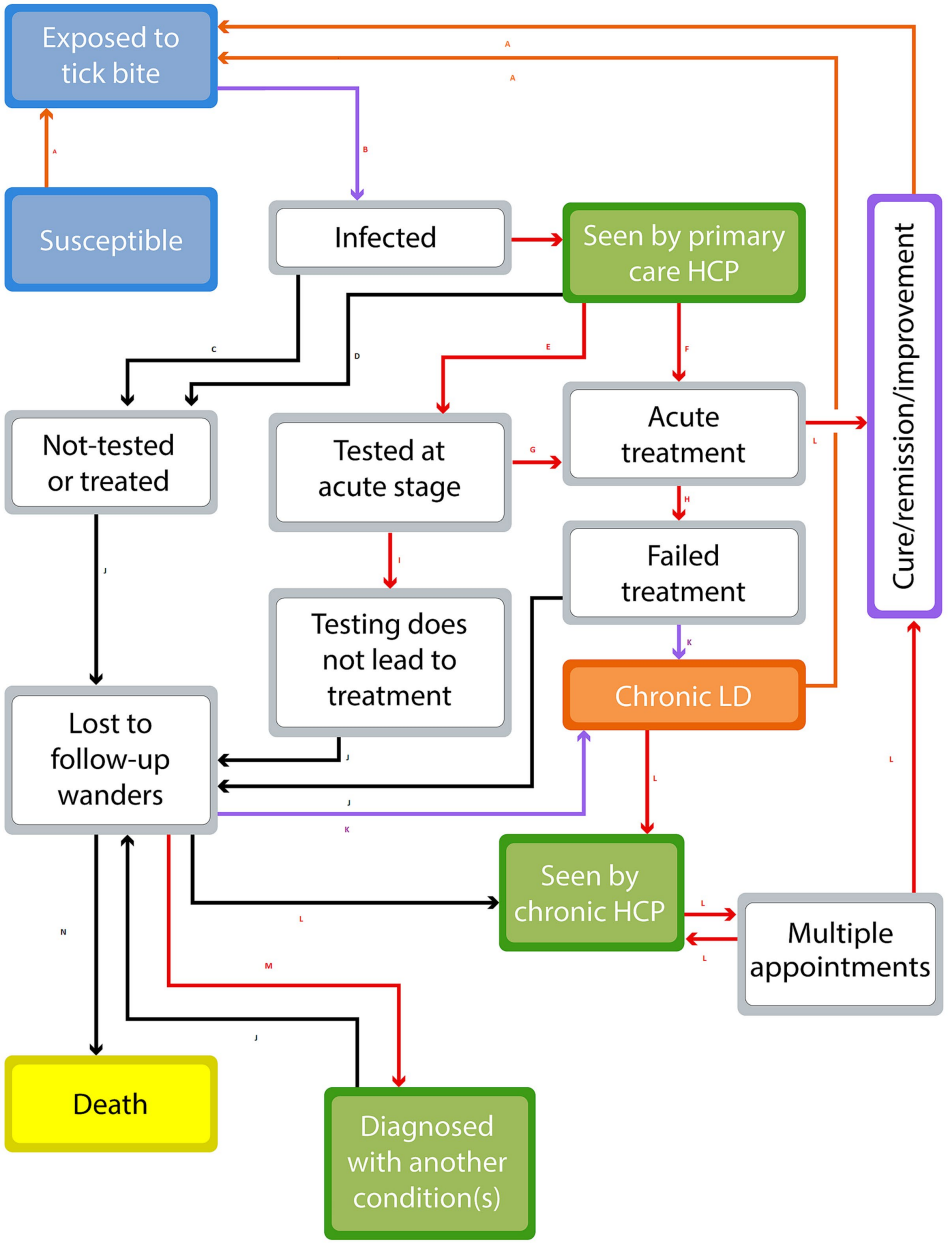
PTLDS patient roadmap modelling. Blue-filled boxes are states with susceptible individuals, and white-filled boxes are states with infected individuals. Purple arrows denote development of chronic symptoms. Green boxes indicate HCP interventions. Black arrows denote all ending up in the lost-to-follow-up state or wandering. The chronic state box encompasses heterogeneous presentations, including PTLDS, untreated/partially treated chronic Lyme, and co-infection sequelae, reflecting real-world clinical complexity and the current limitations in defining chronic tick-borne illness etiology.

Figure 1 outlines the established clinical progression of untreated or delayed-diagnosis Lyme borreliosis, spanning localized, early disseminated, and late disseminated stages (18, 39). This foundational model is included to contextualize the clinical pathways that may precede chronic symptom complexes, as mapped in the novel patient roadmap (Figure 2). Figure 2 illustrates the progression of a Lyme patient’s journey and highlights difficulties in accessing appropriate care and treatment, resulting in high levels of wandering from specialty to specialty. It starts from susceptibility and exposure to a tick-bite, then continues to infection, and progresses through different stages of disease and care. Arrows labeled A to N represent transitions between stages in the patient care and disease management pathway and highlight difficulties in accessing appropriate care. The cost of antibiotic treatment was estimated in Table 1. We are providing this table for information only.

### 3.3 Estimations of the prevalence and lifetime risk of cumulative incidence of PTLDS

The first method was used to calculate point prevalence, while the second one was used to assess lifetime risk or cumulative incidence of PTLDS. The first method utilized involved calculating the incidence rate by considering the number of cases per 100,000 population per year (PPY) reported in prior studies and the average duration of the disease as defined through the questionnaire. A conservative estimate suggests that about 6% of individuals with Lyme disease develop PTLDS. For this calculation, we used the mean current symptom duration at assessment (7.15 years) from our cross-sectional cohort (Table 2). As patients remain symptomatic at the time of survey completion, this value is right-censored and likely underestimates true total disease duration; consequently, the resulting point prevalence estimate should be interpreted as a conservative lower-bound approximation rather than a definitive population measure. The prevalence of the disease can be estimated by multiplying the incidence rate (0.57/100,000 PPY) by the disease duration in years. This results in a prevalence of 0.57 × 7.15 = 4.0755 per 100,000 individuals, equivalent to 0.004% of the general population suffering from PTLDS (point prevalence) (Table 2).

**Table 2:** Comparison of estimations of PTLDS prevalence in Ireland (using two different methods) and the USA (by applying one method).

Method	Country	Method description	Outcome in percent
From the PPY of LD to calculate prevalence	Ireland	Based on an incidence rate of 9.5 acute Lyme disease cases per 100,000 population per year in Ireland (49). Assuming 6% of acute LD cases progress to PTLDS (conservative estimate derived from symptom difference: 27.2% in LD patients vs. 21.2% in controls), the PTLDS incidence is 0.57 per 100,000 PPY. Multiplying by the average PTLDS duration of 7.15 years yields a point prevalence of 0.57 × 7.15 = 4.0755 per 100,000, or 0.004%.	0.004
Indirect reverse estimation to calculate the cumulative incidence of PTLDS	Ireland	Assumes 10% of the population is exposed to tick bites (62). With 5% of ticks infected (50), and 10% risk of developing LD after an infected tick bite (51), the lifetime infection risk is 0.05% of the population. Of these, 6% develop PTLDS, yielding 0.003% population prevalence. This indirect estimation, based on tick exposure and infection probabilities, yields a lifetime risk of 0.003%. This figure is lower than the point prevalence (0.004%) derived from national incidence data, potentially suggesting underreporting of acute Lyme disease cases or overestimation of the 10% population exposure rate	0.003

PTLDS, chronic Lyme disease; LD, Lyme disease; PPY, population per year.

**Figure 3:**
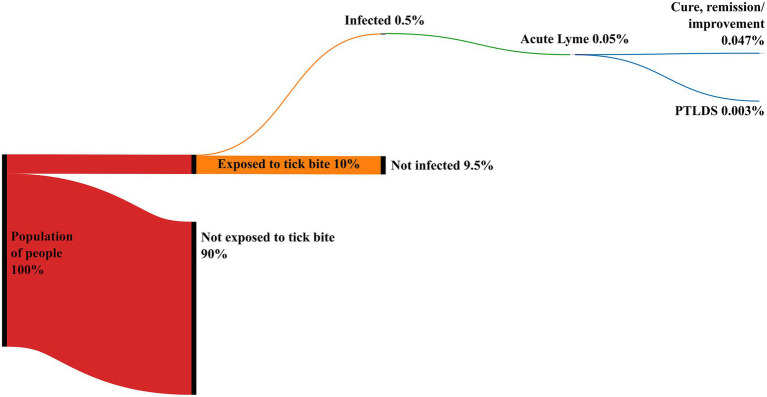
The Sankey diagram illustrates a reverse indirect estimating technique used to determine the occurrence of specific stages in the patient flow model in Ireland.

The second method was a reverse indirect estimating approach of PTLDS and resulted in a prevalence of 0.003% as seen in Figure 3. Figure 3 visually traces the reverse indirect estimation pathway, demonstrating how population-level PTLDS prevalence is back-calculated from tick exposure and progression probabilities. It has been postulated that 10% of individuals have tick bites at some point. Varying this assumption between 5 and 20% would change the prevalence estimate proportionally; formal sensitivity analysis is recommended in future studies. Assuming a 5% *Borrelial* infection rate in ticks and a 10% risk of developing LD after an infected tick bite, the estimated prevalence of LD in Ireland is approximately 3,383 cases. Among these, 6% are expected to develop PTLDS, corresponding to around 203 individuals affected in the population. This estimate translates to a PTLDS prevalence of 0.003% in the general population (Figure 3). Based on this, approximately 13.3% of the Irish population is likely exposed to tick bites. Considering the Irish population of 5,085,375, the number of people bitten by ticks is estimated to be about 33,830. These calculations highlight that the reported incidence is likely an underestimate due to underreporting and limited surveillance, making these figures the lowest possible estimates for Ireland (Figure 3). More details of prevalence estimation calculations are provided in the Supplementary material 2. A one-way sensitivity analysis (Supplementary material 2, Supplementary Table S2.3.5) indicates that PTLDS prevalence estimates range from 0.0002% to 0.081% under plausible variations in tick exposure, Borrelia prevalence, LD development risk, and PTLDS progression parameters. The base-case estimate of 0.003% should therefore be interpreted as a conservative lower-bound approximation.

## 4 Discussion

In the current study, two methods were used to estimate the prevalence of PTLDS in Ireland: the first calculated point prevalence by multiplying the incidence rate of PTLDS (derived from Lyme disease incidence and a conservative 6% PTLDS development rate) by the average disease duration, resulting in 0.004%, which corresponds to approximately 203 people in Ireland. The second method employed a reverse indirect estimation based on the proportion of the population exposed to tick bites, *Borrelial* infection rates, and PTLDS development, yielding a similar prevalence estimate of 0.003%. However, the estimate from the first method likely underestimates the true number of PTLDS cases, as the analyzed study cohort included 210 PTLDS patients, suggesting more comprehensive surveillance is needed. Further studies should focus on improving LD and PTLDS case reporting and surveillance to capture more accurate incidence and prevalence data. Additionally, large-scale, population-based cohort studies with standardized diagnostic criteria for PTLDS could better characterize disease duration and outcomes. Research into risk factors influencing PTLDS development, including tick exposure dynamics, *Borrelial* strain variability, and host susceptibility, would also refine prevalence estimates and guide prevention strategies.

In total, 40.88 M USD were spent in Germany (0.51 USD per capita, 80.59 M), 23.12 M USD in the Netherlands (1.36 USD per capita, 17.08 M), 735,550 USD in Scotland (0.14 USD per capita, population = 5.40 M), and up to 786 M USD in the US (2.41 USD per capita, 326.63 M) were estimated by six studies that evaluated the economic burden from a societal perspective (27). Adopting the PTLDS-based diagnostic methodology is anticipated to result in a 40% reduction in estimated healthcare expenses, as seen by other innovative approaches that include accurate LD testing (54–56). In Belgium and the Netherlands, the burden cost is predicted to be 5.59 M EUR for LD patients; for PTLDS patients, the cost is anticipated to be 26.7 times higher, or 149.8 M EUR (25, 26). Six papers (27) evaluated the financial impact from a social standpoint. They assessed a noteworthy yearly national economic effect of up to 786 M USD in the US, 40.88 M USD in Germany, 23.12 M USD in the Netherlands, and 735,550 USD in Scotland. This work and others (26, 28, 29) have also emphasized the substantial economic burden that LD alone bears; the impact of PTLDS must be factored into these estimates. For instance, the US (30) estimates that the yearly cost of diagnosed LD is between 345 and 968 M USD, which does not account for PTLDS (25–30, 57). On average, individuals with Lyme disease are predicted to incur 2,968 USD more in costs compared to matched controls, resulting in an annual healthcare system expenditure of approximately 1 B USD (57). Other estimates have been higher. With corrections for inflation, a 2006 study found that the mean annual cost of late Lyme disease per patient was 20,502 USD (58, 59). Regarding our study’s data, real data-based conclusions are hard to draw, mainly because of uncertainties regarding prevalence estimates, and we can consider that our data is a preliminary step towards a better assessment of the costs of the disease in Ireland. For now, data from our site is not sufficient to draw a definitive conclusion. While cost figures from Belgium, the Netherlands, Germany, and the US provide contextual benchmarks, they are not adjusted for differing healthcare financing, reimbursement structures, or case-mix. These comparisons are illustrative only and underscore the urgent need for Ireland-specific, standardized economic evaluations.

Lyme disease patients often spend years searching for a diagnosis, as reflected in our patient roadmap data. The recommendation for ‘early detection’ specifically targets acute Lyme disease, which represents the optimal window for targeted antibiotic therapy to prevent progression to chronic sequelae. In practice, GPs should be supported with standardized training to recognize early localized manifestations (e.g., erythema migrans, acute neurologic or cardiac signs), apply appropriate two-tier serologic or direct detection testing, and establish clear referral pathways to Infectious Diseases or specialized tick-borne clinics. We acknowledge that for established PTLDS or chronic tick-borne illness, no single specialty currently serves as a definitive clinical home in Ireland. The observed pattern of patients seeing an average of 17 different physicians underscores the absence of a coordinated multidisciplinary care model. Future policy must address this fragmentation by developing integrated referral frameworks and clarifying specialist responsibilities for chronic symptom management. As a stakeholder, the healthcare system is interested in saving and using unnecessary resources for different purposes, and further investigations into better access to care should be conducted. In this context, standardized further training is essential, especially for GPs, so that this disease can be detected early and clear referral pathways can be established. Such a programme needs to be further developed. The earlier the treatment can be provided, the lower the treatment costs and long-term damage, such as long-term incapacity to work. Barriers to developing better GP guidelines include scientific debates around Lyme disease. However, in practice, GPs should be better encouraged to diagnose the disease and refer it to a specialist.

The prevalence estimates of 0.003–0.004% are exploratory and model-dependent rather than definitive. Their comparatively low magnitude likely reflects Ireland’s narrower surveillance case definitions, passive reporting systems, conservative modeling assumptions, and substantial underascertainment of non-neurological and chronic PTLDS presentations common in other European jurisdictions. Further research into levels of prevalence in the Irish population is needed. More centralized laboratory data shared nationally or compulsory reporting of the results by healthcare professionals to the Irish Health Service Executive would help to assess the scale and impact of LD better by accounting for other forms of LD, not just neuroborreliosis. Additionally, the rate of those who are asymptomatic following an infected tick bite and who do not develop acute illness or chronic symptoms is not well studied and could be better researched.

Climate change is already affecting vector-borne disease transmission and spread, and its impacts are likely to worsen (60). The issue of LD and other tick-borne infections is unlikely to decrease in Ireland, as the European Climate and Health Observatory warns us that warmer temperatures have allowed many disease-carrying vectors to expand their distribution northwards and to higher altitudes in Europe (61). This ecological shift is projected to increase human–tick contact rates, thereby elevating both acute Lyme disease incidence and the downstream prevalence of PTLDS over the coming decades. However, Ireland’s current passive surveillance systems and fragmented diagnostic reporting are ill-equipped to capture these emerging trends, leaving critical gaps in epidemiological forecasting and healthcare resource allocation. To mitigate the anticipated rise in chronic tick-borne disease burden, proactive policy interventions are urgently required. These must include the integration of climate-informed vector monitoring into national public health frameworks, standardized mandatory reporting of all LD/PTLDS manifestations, and the development of adaptive clinical pathways that account for shifting geographic risk profiles. Without such measures, the compounding effects of climate-driven vector expansion will likely outpace existing healthcare capacity, further delaying diagnosis and escalating long-term patient and societal costs.

## 5 Study strengths and limitations

Although our study provides some data where none is recorded, more Irish data is still needed to fill the gaps and arrive at a robust cost analysis. Robust Irish prevalence studies must be conducted, considering any testing and disease recognition issues. Publications from international literature were used to fill knowledge gaps. Moreover, an assumption was made about the number of people exposed to tick bites. An estimated value of 10% is suggested in the current paper. If the proportions were significantly different, then the estimated proportion of people suffering from PTLDS would also differ. Due to limitations in establishing prevalence estimates, a further cost analysis based on actual data was limited. Furthermore, while a univariate sensitivity analysis was performed for the reverse indirect estimation method (Method 2; Supplementary material 2, Section S2.3.5), no equivalent quantitative sensitivity analysis was conducted for the point prevalence method (Method 1). This omission reflects the reliance on right-censored duration data (7.15 years measured at assessment), which limits the ability to model uncertainty around total disease duration. Future work should incorporate probabilistic sensitivity analyses (e.g., Monte Carlo simulation) for both methods to generate formal uncertainty intervals around prevalence estimates. This study was conducted at a single tertiary infectious diseases clinic using a convenience sample, which inherently limits external validity and generalizability to the broader Irish population. Recruitment from a single specialty TBI clinic limits representativeness, as patients likely represent a subset with prolonged diagnostic delays, higher symptom burden, or treatment-refractory courses. The cross-sectional, questionnaire-based design lacks a matched control group, and self-reported diagnoses and symptom durations were not independently validated through clinical or laboratory records. Furthermore, while the patient roadmap was constructed using existing literature and expert consensus, it has not yet undergone formal qualitative validation via patient interviews or focus groups. Future research should prioritize multi-center recruitment, clinical validation of self-reported data, and mixed-methods qualitative validation to refine care pathways and improve population representativeness. Last, but not least, the absence of universally standardized diagnostic criteria remains a recognized limitation across PTLDS research. Given the single-site design and cross-sectional nature of the patient questionnaire, the descriptive care utilization and cost estimates should be interpreted as preliminary benchmarks rather than population-level measures. The disease modeling and prevalence estimates rely on conservative assumptions and published international parameters, reflecting a gap-filling approach necessitated by the lack of Irish surveillance data. Future research should prioritize multi-center recruitment, prospective longitudinal design, and independent validation of each modeled component. Our cohort strictly adhered to the >6 months post-treatment symptom criterion, ensuring that all 210 participants represent a homogeneous PTLDS population rather than a broader CLD or mixed tick-borne cohort. This standardization strengthens the internal validity of our care-utilization and economic burden estimates, though it may limit generalizability to patients with untreated or polymicrobial chronic presentations.

## 6 Conclusion

Current estimates are preliminary and should not be interpreted as definitive burden measures. The results from this study highlight the negative long-term consequences of contracting LD and co-infections for patients, with a loss of capacity for employment for over two-thirds of those patients. In addition, the lack of proper pathways to care and debates over chronic symptoms put patients on a difficult journey within the healthcare system, as seen in the patient roadmap from our study, with many left wandering from specialty to specialty for their care over long periods of time, averaging over 7 years. Actual data is missing to provide a reliable Irish data-based estimate of the PTLDS burden, but our estimate of the lifetime risk of cumulative incidence using indirect reverse estimation is at 0.003% when gaps were filled where needed, with a prevalence estimate at 0.004%. In an era of climate change, ticks and TBIs have been highlighted as major health concerns, as higher temperatures are likely to increase the number of ticks, and therefore the number of infections. This article calls for better monitoring of the disease in Ireland and to include monitoring for forms of testing other than cerebrospinal fluid for neuroborreliosis, and including PTLDS to inform policy at the national level. We also call for the provision of better pathways to care, inclusive of patient needs, and with feedback from patients, so that this public health concern can be properly assessed and addressed. In the absence of a change, it is clear that patients are left to deal with the gaps in the system and their cost in terms of health and financial outcomes. Definitive assessment requires multi-center, population-based studies with standardized diagnostic criteria, active surveillance, and comprehensive health-economic modeling.

## Data Availability

The original contributions presented in the study are included in the article/Supplementary material, further inquiries can be directed to the corresponding author.

## References

[1] Dong Y Zhou G Cao W Xu X Zhang Y Ji Z . Global seroprevalence and sociodemographic characteristics of *Borrelia burgdorferi sensu lato* in human populations: a systematic review and meta-analysis. BMJ Glob Health. (2022) 7:e007744. doi: 10.1136/bmjgh-2021-007744 PMC9185477 35697507

[2] Burn L Pilz A Vyse A Rabá AVG Angulo FJ Tran TMP . Seroprevalence of Lyme Borreliosis in Europe: results from a systematic literature review (2005–2020). Vector Borne Zoonotic Dis. (2023) 23:195–220. doi: 10.1089/vbz.2022.0069, 37071401 PMC10122246

[3] Tokarska-Rodak M Plewik D Kozioł-Montewka M Szepeluk A Paszkiewicz J. Ryzyko zakażeń zawodowych *Borrelia burgdorferi* u pracowników leśnictwa i rolników. Med Pr. (2014) 65:109–17. 24834698 10.13075/mp.5893.2014.017

[4] Davidson A Davis J Brestrich G Moisi JC Jodar L Stark JH. Lyme Borreliosis incidence across Europe, 2015-2023: a surveillance-based review and analysis. Vector Borne Zoonotic Dis. (2025) 25:569–79. doi: 10.1177/15303667251363125 40711909 PMC12629682

[5] Burn L Vyse A Pilz A Tran TMP Fletcher MA Angulo FJ . Incidence of Lyme Borreliosis in Europe: a systematic review (2005–2020). Vector Borne Zoonotic Dis. (2023) 23:172–94. doi: 10.1089/vbz.2022.0070, 37071407 PMC10122234

[6] Parm Ü Niitvägi E Beljaev K Aro T Aotäht E Raska K . Puukborrelioos Saaremaal. Eesti Arst. (2015). 94:203–210. doi: 10.15157/ea.v0i0.12011

[7] Wormser GP Dattwyler RJ Shapiro ED Halperin JJ Steere AC Klempner MS . The clinical assessment, treatment, and prevention of Lyme disease, human granulocytic Anaplasmosis, and Babesiosis: clinical practice guidelines by the Infectious Diseases Society of America. Clin Infect Dis. (2006) 43:1089–134. doi: 10.1086/508667, 17029130

[8] Marques A. Chronic Lyme disease: a review. Infect Dis Clin N Am. (2008) 22:341–60. doi: 10.1016/j.idc.2007.12.011, 18452806 PMC2430045

[9] Aucott JN Rebman AW Crowder LA Kortte KB. Post-treatment Lyme disease syndrome symptomatology and the impact on life functioning: is there something here? Qual Life Res. (2013) 22:75–84. doi: 10.1007/s11136-012-0126-6, 22294245 PMC3548099

[10] Ursinus J Vrijmoeth HD Harms MG Tulen AD Knoop H Gauw SA . Prevalence of persistent symptoms after treatment for Lyme borreliosis: a prospective observational cohort study. Lancet Reg Health. (2021) 6:100142. doi: 10.1016/j.lanepe.2021.100142, 34557833 PMC8454881

[11] Xi D Thoma A Rajput-Ray M Madigan A Avramovic G Garg K . A longitudinal study of a large clinical cohort of patients with Lyme disease and tick-borne co-infections treated with combination antibiotics. Microorganisms. (2023) 11:2152. doi: 10.3390/microorganisms11092152, 37763996 PMC10536678

[12] Lantos PM Rumbaugh J Bockenstedt LK Falck-Ytter YT Aguero-Rosenfeld ME Auwaerter PG . Clinical practice guidelines by the Infectious Diseases Society of America (IDSA), American Academy of Neurology (AAN), and American College of Rheumatology (ACR): 2020 guidelines for the prevention, diagnosis and treatment of Lyme disease. Clin Infect Dis. (2020) 72:1–8. doi: 10.1212/WNL.0000000000011151 33483734

[13] Kaplan RF Trevino RP Johnson GM Levy L Dornbush R Hu LT . Cognitive function in post-treatment Lyme disease do additional antibiotics help? Neurology. (2003) 60:1916–22. doi: 10.1212/01.WNL.0000068030.26992.25, 12821733

[14] Shor S Green C Szantyr B Phillips S Liegner K Burrascano J . Chronic Lyme disease: an evidence-based definition by the ILADS working group. Antibiotics. (2019) 8:269. doi: 10.3390/antibiotics8040269, 31888310 PMC6963229

[15] Rebman AW Yang T Aucott JN. Symptom heterogeneity and patient subgroup classification among US patients with post-treatment Lyme disease: an observational study. BMJ Open. (2021) 11:e040399. doi: 10.1136/bmjopen-2020-040399, 33441353 PMC7812114

[16] Fiore AE. Progress toward understanding infection-associated chronic conditions and illnesses. Emerg Infect Dis. (2025) 31:1–2. doi: 10.3201/eid3114.251187, 41570177 PMC12829550

[17] DeLong A Hsu M Kotsoris H. Estimation of cumulative number of post-treatment Lyme disease cases in the US, 2016 and 2020. BMC Public Health. (2019) 19:352. doi: 10.1186/s12889-019-6681-9, 31014314 PMC6480773

[18] Cardenas-de la Garza JA De la Cruz-Valadez E Ocampo-Candiani J Welsh O. Clinical spectrum of Lyme disease. Eur J Clin Microbiol Infect Dis. (2019) 38:201–8. doi: 10.3389/fmed.2020.00265 30456435

[19] Marques A. Persistent symptoms after treatment of Lyme disease. Infect Dis Clin N Am. (2022) 36:621–38. doi: 10.1016/j.idc.2022.04.004, 36116839 PMC9494579

[20] Steere AC Sikand VK Schoen RT Nowakowski J. Asymptomatic infection with *Borrelia burgdorferi*. Clin Infect Dis. (2003) 37:528–32. doi: 10.1086/376914, 12905137

[21] Hoeve-Bakker BJA van den Berg OE Doppenberg HS van der Klis FRM van den Wijngaard CC . Seroprevalence and risk factors of Lyme borreliosis in the Netherlands: a population-based cross-sectional study. Microorganisms. (2023) 11:1081. doi: 10.3390/microorganisms11041081, 37110504 PMC10143428

[22] Zhioua E Gern L Aeschlimann A Sauvain MJ der Linden SV Fahrer H. Longitudinal study of Lyme borreliosis in a high risk population in Switzerland. Parasite. (1998) 5:383–6. doi: 10.1051/parasite/1998054383, 9879563

[23] Davidsson M. The financial implications of a well-hidden and ignored chronic Lyme disease pandemic. Healthcare. (2018) 6:16. doi: 10.3390/healthcare6010016, 29438352 PMC5872223

[24] Willems R Verhaeghe N Perronne C Borgermans L Annemans L. Cost of illness in patients with post-treatment Lyme disease syndrome in Belgium. Eur J Public Heal. (2023) 33:668–74. doi: 10.1093/eurpub/ckad045, 36972275 PMC10393486

[25] van den Wijngaard CC Hofhuis A Wong A Harms MG de Wit GA Lugnér AK . The cost of Lyme borreliosis. Eur J Pub Health. (2017) 27:538–47. doi: 10.1093/eurpub/ckw269, 28444236

[26] Mac S Evans GA Patel SN Pullenayegum EM Sander B. Estimating the population health burden of Lyme disease in Ontario, Canada: a microsimulation modelling approach. Can Méd Assoc Open Access J. (2021) 9:E1005–12. doi: 10.9778/cmajo.20210024 PMC8598239 34785530

[27] Mac S da Silva SR Sander B. The economic burden of Lyme disease and the cost-effectiveness of Lyme disease interventions: a scoping review. PLoS One. (2019) 14:e0210280. doi: 10.1371/journal.pone.0210280 30608986 PMC6319811

[28] Mac S Evans G Pullenayegum E Patel SN Sander B. Healthcare costs and outcomes associated with laboratory-confirmed Lyme disease in Ontario, Canada: a population-based cohort study. PLoS One. (2023) 18:e0286552. doi: 10.1371/journal.pone.0286552, 37347742 PMC10286989

[29] Rogalska AM Pawełczyk O Solarz K Holecki T. What are the costs of diagnostics and treatment of Lyme borreliosis in Poland? Front Public Health. (2021) 8:599239. doi: 10.3389/fpubh.2020.599239 33537276 PMC7848162

[30] Hook SA Jeon S Niesobecki SA Hansen AP Meek JI Bjork JKH . Economic burden of reported Lyme disease in high-incidence areas, United States, 2014–2016. Emerg Infect Dis. (2022) 28:1170–9. doi: 10.3201/eid2806.211335 35608612 PMC9155891

[31] Aucott JN Yang T Yoon I Powell D Geller SA Rebman AW. Risk of post-treatment Lyme disease in patients with ideally-treated early Lyme disease: a prospective cohort study. Int J Infect Dis. (2022) 116:230–7. doi: 10.1016/j.ijid.2022.01.033, 35066160

[32] Trouillas P Franck M. Complete remission in paralytic late tick-borne neurological disease comprising mixed involvement of *Borrelia*, *Babesia*, *Anaplasma*, and *Bartonella*: use of long-term treatments with antibiotics and Antiparasitics in a series of 10 cases. Antibiotics. (2023) 12:1021. doi: 10.3390/antibiotics12061021, 37370340 PMC10294829

[33] Xi D Garg K Lambert JS Rajput-Ray M Madigan A Avramovic G . Scrutinizing clinical biomarkers in a large cohort of patients with Lyme disease and other tick-borne infections. Microorganisms. (2024) 12:380. doi: 10.3390/microorganisms12020380, 38399784 PMC10893018

[34] Krkic-Dautovic S Salihbegovic A Dervisevic E Gojak R Hadzovic-Cengic M Duratbegovic D . Clinical manifestations of European borreliosis on the skin in acute, subacute and chronic disease. Mater Sociomed. (2024) 36:33–9. doi: 10.5455/msm.2024.36.33-39 38590600 PMC10999147

[35] Berghoff W. Chronic Lyme disease and co-infections: differential diagnosis. Open Neurol J. (2012) 6:158–78. doi: 10.2174/1874205X01206010158, 23400696 PMC3565243

[36] Swanson SJ Neitzel D Reed KD Belongia EA. Coinfections acquired from *Ixodes* ticks. Clin Microbiol Rev. (2006) 19:708–27. doi: 10.1128/cmr.00011-06, 17041141 PMC1592693

[37] Krause PJ McKay K Thompson CA Sikand VK Lentz R Lepore T . Disease-specific diagnosis of coinfecting tickborne zoonoses: Babesiosis, human granulocytic Ehrlichiosis, and Lyme disease. Clin Infect Dis. (2002) 34:1184–91. doi: 10.1086/339813, 11941544

[38] Diuk-Wasser MA Vannier E Krause PJ. Coinfection by Ixodes tick-borne pathogens: ecological, epidemiological, and clinical consequences. Trends Parasitol. (2016) 32:30–42. doi: 10.1016/j.pt.2015.09.008, 26613664 PMC4713283

[39] Steere AC Coburn J Glickstein L. The emergence of Lyme disease. J Clin Invest. (2004) 113:1093–101. doi: 10.1172/JCI21681, 15085185 PMC385417

[40] Schwartz AM Hinckley AF Mead PS Hook SA Kugeler KJ. Surveillance for Lyme disease — United States, 2008–2015. MMWR Surveill Summ. (2017) 66:1–12. doi: 10.15585/mmwr.ss6622a1, 29120995 PMC5829628

[41] INESSS-Institut National en Sante et en Services Sociaux-Quebec National Institute for Excellence in Health and Social Services-Canada. I national d’excellence en santé et en services sociaux. Rapport rédigé par Geneviève Morrow. Avis: Maladie de Lyme—stades localisé et disséminés. Situation actuelle et accompagnement vers le changement. Available online at: https://www.inesss.qc.ca/fileadmin/doc/INESSS/Rapports/Biologie_medicale/Lyme_PPE/INESSS_Avis_Lyme.pdf (Accessed June 2, 2026).

[42] Bridges JFP de Bekker-Grob EW Hauber B Heidenreich S Janssen E Bast A . A roadmap for increasing the usefulness and impact of patient-preference studies in decision making in health: a good practices report of an ISPOR task force. Value Health. (2023) 26:153–62. doi: 10.1016/j.jval.2022.12.004, 36754539

[43] Kowalski EN Qian G Vanni KMM Sparks JA. A roadmap for investigating preclinical autoimmunity using patient-oriented and epidemiologic study designs: example of rheumatoid arthritis. Front Immunol. (2022) 13:890996. doi: 10.3389/fimmu.2022.890996, 35693829 PMC9175569

[44] Stegemann S Sheehan L Rossi A Barrett A Paudel A Crean A . Rational and practical considerations to guide a target product profile for patient-centric drug product development with measurable patient outcomes – a proposed roadmap. Eur J Pharm Biopharm. (2022) 177:81–8. doi: 10.1016/j.ejpb.2022.06.006, 35718077

[45] Wilson NA Tcheng JE Graham J Drozda JP Jr. Advancing patient safety surrounding medical devices: a health system roadmap to implement unique device identification at the point of care. Méd Devices (Auckl). (2021) 14:411–21. doi: 10.2147/MDER.S339232 34880686 PMC8645947

[46] Ward Z Reynolds R Campbell L Martin NK Harrison G Irving W . Cost-effectiveness of the HepCATT intervention in specialist drug clinics to improve case-finding and engagement with HCV treatment for people who inject drugs in England. Addiction. (2020) 115:1509–21. doi: 10.1111/add.14978, 31984606 PMC10762643

[47] Spronk I Korevaar JC Poos R Davids R Hilderink H Schellevis FG . Calculating incidence rates and prevalence proportions: not as simple as it seems. BMC Public Health. (2019) 19:512. doi: 10.1186/s12889-019-6820-3, 31060532 PMC6501456

[48] Penev DG Laurent E Baron S Diot E Bastides F de Gialluly C . Borréliose de Lyme: recensement des cas adultes hospitalisés en Indre-et-Loire, à partir du PMSI (1999–2006). Rev Epidemiol Sante Publique. (2010) 58:339–47. doi: 10.1016/j.respe.2010.05.003, 20708866

[49] Brestrich G Shafquat M Angulo FJ Davidson A Tan Y Halsby K . Using meta-analysis to estimate the incidence of Lyme borreliosis clinical manifestations in Denmark, Ireland and Sweden based on publicly-available Lyme neuroborreliosis data. Ticks Tick Borne Dis. (2025) 16:102509. doi: 10.1016/j.ttbdis.2025.102509, 40577940

[50] Lambert JS Cook MJ Healy JE Murtagh R Avramovic G Lee SH. Metagenomic 16S rRNA gene sequencing survey of *Borrelia* species in Irish samples of *Ixodes ricinus* ticks. PLoS One. (2019) 14:e0209881. doi: 10.1371/journal.pone.0209881, 30986208 PMC6464168

[51] Hofhuis A van de Kassteele J Sprong H van den Wijngaard CC Harms MG Fonville M . Predicting the risk of Lyme borreliosis after a tick bite, using a structural equation model. PLoS One. (2017) 12:e0181807. doi: 10.1371/journal.pone.0181807 28742149 PMC5524385

[52] Rebman AW Bechtold KT Yang T Mihm EA Soloski MJ Novak CB . The clinical, symptom, and quality-of-life characterization of a well-defined group of patients with posttreatment Lyme disease syndrome. Front Med. (2017) 4:224. doi: 10.3389/fmed.2017.00224 PMC5735370 29312942

[53] Mauri M Elli T Caviglia G Uboldi G Azzi M. RAWGraphs: a visualisation platform to create open outputs. In: Proceedings of the 12th Biannual Conference on Italian SIGCHI Chapter. New York, NY, USA: Association for Computing Machinery; (CHItaly ‘17). 28:1–28:5. doi: 10.1145/3125571.3125585

[54] Worldmeters (2024). Available online at: https://www.worldometers.info/world-population/ireland-population/ (Accessed June 2, 2026).

[55] Geebelen L Devleesschauwer B Lernout T Tersago K Parmentier Y Oyen HV . Lyme borreliosis in Belgium: a cost-of-illness analysis. BMC Public Health. (2022) 22:2194. doi: 10.1186/s12889-022-14380-6 36443755 PMC9703731

[56] Pradelli L Pinciroli M Houshmand H Grassi B Bonelli F Calleri M . Comparative cost and effectiveness of a new algorithm for early Lyme disease diagnosis: evaluation in US, Germany, and Italy. Clin Outcomes Res. (2021) 13:437–51. doi: 10.2147/CEOR.S306391 PMC8165099 34079307

[57] Mac S Bahia S Simbulan F Pullenayegum EM Evans GA Patel SN . Long-term sequelae and health-related quality of life associated with Lyme disease: a systematic review. Clin Infect Dis. (2019) 71:440–52. doi: 10.1093/cid/ciz1158 PMC7353842 31773171

[58] Zhang X Meltzer MI Peña CA Hopkins AB Wroth L Fix AD. Economic impact of Lyme disease. Emerg Infect Dis. (2006) 12:653–60. doi: 10.3201/eid1204.050602, 16704815 PMC3294685

[59] Johnson L. Insurance and Lyme disease: a problem of displaced costs [Internet]. LymeDisease.org. (2013). Available online at: https://www.lymedisease.org/wp-content/uploads/2015/02/Chart-book-11.14.13-FINAL-with-correction.pdf (Accessed June 2, 2026).

[60] Rocklöv J Dubrow R. Climate change: an enduring challenge for vector-borne disease prevention and control. Nat Immunol. (2020) 21:479–83. doi: 10.1038/s41590-020-0648-y, 32313242 PMC7223823

[61] European Climate and Health Observatory. (2026). Vectorborne diseases. Copenhagen: European Environment Agency. Available online at: https://climate-adapt.eea.europa.eu/en/observatory/evidence/health-effects/vector-borne-diseases (Accessed June 2, 2026).

[62] Steere AC Taylor E McHugh GL Logigian EL. The overdiagnosis of Lyme disease. JAMA. (1993) 269:1812–6. doi: 10.1001/jama.1993.03500140064037 8459513

