# Supplementary material for: Patient roadmap and economic burden of chronic tick-borne illness and post-treatment Lyme disease syndrome in Ireland, and public health issues arising
Source: Front Public Health. 2026 Jun 10;14:1667043. doi: 10.3389/fpubh.2026.1667043 (PMC13290999; doi:10.3389/fpubh.2026.1667043)
Supplement: Supplementary file 2 [file Supplementary_file_2.docx]

**Supplementary Material S2: Detailed Prevalence Estimation Methodology**

**Manuscript Title:** Patient Roadmap and Economic Burden of Post-Treatment Lyme Disease Syndrome (PTLDS) and Other Tick-Borne Co-Infections in Ireland, and Public Health Issues Arising

**Corresponding Author:** jlambert@mater.ie

**S2.1 Overview of Estimation Approaches**

Two complementary methodologies were employed to estimate the burden of PTLDS in Ireland:

| **Method** | **Purpose** | **Key Output** |
| --- | --- | --- |
| **Method 1: Point Prevalence** | Estimate the proportion of the Irish population living with PTLDS at a given time | Point prevalence (% of population) |
| **Method 2: Reverse Indirect Estimation** | Model the cumulative lifetime risk of developing PTLDS based on tick exposure pathways | Cumulative incidence/lifetime risk (% of population) |

Both methods rely on conservative, literature-derived parameters. All calculations assume a static Irish population of **5,085,375** (CSO, 2022).

**S2.2 Method 1: Point Prevalence Calculation**

**S2.2.1 Formula Derivation**

The standard epidemiological relationship for steady-state conditions:

Prevalence=Incidence Rate×Average Disease DurationPrevalence=Incidence Rate×Average Disease Duration

Where:

- Prevalence = proportion of population with PTLDS at a point in time
- Incidence Rate = new PTLDS cases per 100,000 population per year (PPY)
- Average Disease Duration = mean years lived with PTLDS (from cohort data)

**S2.2.2 Parameter Inputs and Justifications**

| **Parameter** | **Value** | **Source/Justification** | **Range for Sensitivity** |
| --- | --- | --- | --- |
| **Acute LD incidence (Ireland)** | 9.5 /100,000 PPY | Brestrich et al. (49); meta-analysis of neuroborreliosis data | 5.0 – 15.0 /100,000 PPY |
| **PTLDS progression rate** | 6% | Conservative estimate: difference between LD cohort (27.2%) and population controls (21.2%) for chronic symptoms (10,53) | 3% – 15% |
| **Derived PTLDS incidence** | 0.57 /100,000 PPY | Calculated: 9.5 × 0.06 | 0.15 – 2.25 /100,000 PPY |
| **Average PTLDS duration** | 7.15 years | Cohort data (Table 1); excludes patients with symptoms <6 months | 5.0 – 10.0 years |
| **Irish population** | 5,085,375 | Central Statistics Office Ireland, 2022 | Fixed |

**S2.2.3 Step-by-Step Calculation**

**Step 1:** Calculate PTLDS incidence rate

PTLDS Incidence = LD Incidence × Progression Rate = 9.5 per 100,000 PPY × 0.06 = 0.57 per 100,000 PPY

**Step 2:** Apply prevalence formula

Point Prevalence = 0.57 per 100,000 PPY × 7.15 years = 4.0755 per 100,000 population

**Step 3:** Convert to percentage

Prevalence (%) = (4.0755 / 100,000) × 100 = 0.0040755% ≈ **0.004%**

**Step 4:** Estimate absolute case count

Estimated Cases = 5,085,375 × 0.000040755 ≈ **207 individuals**

**S2.2.4 Key Assumptions and Limitations**

1. **Steady-state assumption**: Incidence and duration are constant over time
2. **No mortality differential**: PTLDS does not significantly affect life expectancy
3. **Case definition consistency**: PTLDS defined as symptoms >6 months post-treatment
4. **Under-ascertainment**: Acute LD incidence (9.5/100,000) likely underestimates true incidence due to passive surveillance

**S2.3 Method 2: Reverse Indirect Estimation**

**S2.3.1 Conceptual Framework**

This method models the sequential probability pathway from tick exposure to PTLDS:

General Population

↓

[Step 1] Tick Bite Exposure (Probability: P₁)

↓

[Step 2] Tick Infected with Borrelia (Probability: P₂)

↓

[Step 3] Development of Acute Lyme Disease (Probability: P₃)

↓

[Step 4] Progression to PTLDS (Probability: P₄)

↓

Estimated PTLDS Cases in Population

**S2.3.2 Formula Derivation**

**Forward pathway** (population → PTLDS cases):

N_PTLDS​=_N_pop_​×P_1_​×P_2_​×P_3_​×P_4​_

**Reverse pathway** (PTLDS cases → estimated tick exposures):

Ntick_exposed=N_PTLDS​/_(P_2_​×P_3_​×P_4_)​

Where:

- N_pop_ = total Irish population
- P_1_​ = probability of lifetime tick bite exposure
- P_2_​ = probability tick carries *Borrelia*
- P_3_​ = probability of developing acute LD after infected bite
- P_4_ = probability of progressing from acute LD to PTLDS

**S2.3.3 Parameter Inputs and Justifications**

| **Parameter** | **Symbol** | **Base Value** | **Source/Justification** | **Plausible Range** |
| --- | --- | --- | --- | --- |
| **Lifetime tick bite exposure** | P₁ | 10% | Steer et al. (51); conservative estimate for temperate European regions | 5% – 25% |
| **Borrelia prevalence in Irish ticks** | P₂ | 5% | Lambert et al. (53); metagenomic survey of *I. ricinus* in Ireland | 2% – 15% |
| **LD development after infected bite** | P₃ | 10% | Hofhuis et al. (54); structural equation model of tick bite outcomes | 6.7% – 14.4% |
| **PTLDS progression after acute LD** | P₄ | 6% | Conservative differential: 27.2% LD cohort vs. 21.2% controls (10,53) | 3% – 15% |
| **Combined pathway probability** | P₁×P₂×P₃×P₄ | 0.0003 (0.03%) | Calculated: 0.10 × 0.05 × 0.10 × 0.06 | 0.00002 – 0.00135 |

**S2.3.4 Step-by-Step Calculation**

**Step 1:** Calculate combined probability of PTLDS development

**Step 1:** Calculate combined probability of PTLDS development

P_combined = P_1 × P_2 × P_3 × P_4 = 0.10 × 0.05 × 0.10 × 0.06 = 0.00003 = **0.003%**

**Step 2:** Estimate absolute PTLDS cases

N_PTLDS = 5,085,375 × 0.00003 = **153 individuals**

**Step 3:** Reverse calculation – estimate tick-exposed population

N_tick_exposed = N_PTLDS / (P_2 × P_3 × P_4) = 153 / (0.05 × 0.10 × 0.06) = 153 / 0.0003 = **510,000 individuals**

**Step 4:** Derive implied exposure prevalence

P_1_implied = 510,000 / 5,085,375 = 0.1003 ≈ **10.0%** *(Validates consistency of forward/reverse calculations)*

**Step 5:** Express as population prevalence

PTLDS Prevalence = **0.003%** of Irish population

**S2.3.5 Sensitivity Analysis: Parameter Variation Impact**

| **Scenario** | **P₁** | **P₂** | **P₃** | **P₄** | **Combined P** | **Estimated Cases** | **Prevalence (%)** |
| --- | --- | --- | --- | --- | --- | --- | --- |
| **Base (conservative)** | 10% | 5% | 10% | 6% | 0.003% | 153 | 0.003 |
| Low exposure | 5% | 5% | 10% | 6% | 0.0015% | 76 | 0.0015 |
| High exposure | 25% | 5% | 10% | 6% | 0.0075% | 382 | 0.0075 |
| High tick infection | 10% | 15% | 10% | 6% | 0.009% | 458 | 0.009 |
| High LD risk | 10% | 5% | 14.4% | 6% | 0.0043% | 219 | 0.0043 |
| High PTLDS progression | 10% | 5% | 10% | 15% | 0.0075% | 382 | 0.0075 |
| **Upper bound (all high)** | 25% | 15% | 14.4% | 15% | 0.081% | 4,119 | 0.081 |
| **Lower bound (all low)** | 5% | 2% | 6.7% | 3% | 0.0002% | 10 | 0.0002 |

*Note: Extreme scenarios (all high/all low) are biologically implausible but illustrate parameter sensitivity.*

**S2.4 Comparison of Methods and Interpretation**

| **Feature** | **Method 1: Point Prevalence** | **Method 2: Reverse Indirect** |
| --- | --- | --- |
| **Primary input** | Reported LD incidence + cohort duration | Tick ecology + progression probabilities |
| **Key assumption** | Steady-state epidemiology | Independent sequential probabilities |
| **Strengths** | Grounded in surveillance data; transparent duration estimate | Explicitly models exposure pathway; useful for forecasting |
| **Limitations** | Depends on under-ascertained LD incidence; assumes constant duration | Compounds uncertainty across four parameters; assumes independence |
| **Output** | 0.004% (≈207 cases) | 0.003% (≈153 cases) |
| **Interpretation** | Current burden estimate | Lifetime cumulative risk estimate |

**Reconciliation**: The close agreement between methods (0.003% vs. 0.004%) despite different inputs suggests internal consistency. The slight discrepancy may reflect:

- Under-reporting of acute LD cases (affecting Method 1)
- Conservative tick exposure assumption (affecting Method 2)

**S2.5 Critical Assumptions and Their Implications**

**S2.5.1 Assumption of Parameter Independence**

The reverse model assumes P1, P2​, P3​, and P4​ are independent. In reality:

- Tick attachment duration affects both P2​ (infection likelihood) and P3​ (LD development)
- Host immunity may influence both P3 and P4
- *Implication*: True combined probability may differ from simple product; results should be interpreted as first-order approximations.

**S2.5.2 Conservative Parameter Selection**

All base-case parameters were intentionally selected at lower bounds of published ranges to avoid overestimation. This means:

- **Reported prevalence estimates (0.003–0.004%) represent minimum plausible values**
- True burden is likely higher, potentially substantially so if any parameter is underestimated

**S2.5.3 Static Population and Time-Invariant Parameters**

Calculations assume:

- No population growth or demographic shift
- No temporal trends in tick density, *Borrelia* prevalence, or climate effects
- *Implication*: Estimates reflect a snapshot; climate change and land-use changes may increase future risk (see Discussion, Lines 341–346).

**S2.5.4 Case Definition Consistency**

PTLDS defined uniformly as:

Persistent or recurring symptoms >6 months following completion of recommended antibiotic therapy for Lyme disease, per prevailing clinical frameworks (7,9,14–16).

This excludes:

- Patients with chronic symptoms but no prior confirmed LD diagnosis
- Patients with co-infections but no *Borrelia* exposure
- *Implication*: Estimates capture only a subset of the broader chronic tick-borne illness burden.
